# GraphFind: enhancing graph searching by low support data mining techniques

**DOI:** 10.1186/1471-2105-9-S4-S10

**Published:** 2008-04-25

**Authors:** Alfredo Ferro, Rosalba Giugno, Misael Mongiovì, Alfredo Pulvirenti, Dmitry Skripin, Dennis Shasha

**Affiliations:** 1 Dipartimento di Matematica e Informatica, Università di Catania, Catania, 95125, Italy; 2 Dipartimento di Scienze Biomediche, Università di Catania, Catania, 95125, Italy; 3 Courant Institute of Mathematical Sciences, New York University, New York, 10012, USA

## Abstract

**Background:**

Biomedical and chemical databases are large and rapidly growing in size. Graphs naturally model such kinds of data. To fully exploit the wealth of information in these graph databases, a key role is played by systems that search for all exact or approximate occurrences of a query graph. To deal efficiently with graph searching, advanced methods for indexing, representation and matching of graphs have been proposed.

**Results:**

This paper presents GraphFind. The system implements efficient graph searching algorithms together with advanced filtering techniques that allow approximate search. It allows users to select candidate subgraphs rather than entire graphs. It implements an effective data storage based also on low-support data mining.

**Conclusions:**

GraphFind is compared with Frowns, GraphGrep and gIndex. Experiments show that GraphFind outperforms the compared systems on a very large collection of small graphs. The proposed low-support mining technique which applies to any searching system also allows a significant index space reduction.

## Background

**Figure 1:**
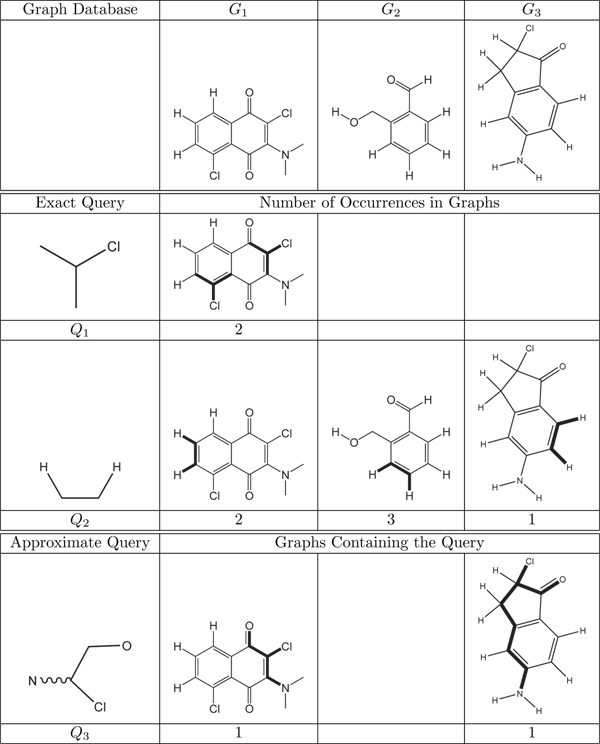
**Querying a database of molecules**. Graphs represent molecules. During the match process, edge information is ignored. Query occurrences are shown in bold. For *Q*_2_ since query matches overlap, only one occurrence in each molecule is depicted. The number of occurrences is also given. Molecular descriptions include hydrogen atoms for search accuracy. In a context where hydrogen atoms are not considered, query *Q*_2_ is present 11 times in *G*_1_, 6 in *G*_2_ and 10 in *G*_3_. The approximate query specifies any path of an unspecified length between atoms *C* and *N*. Approximate queries may also contain atoms with unknown label (they match any atom).

Application domains such as bioinformatics and cheminformatics represent data as graphs where nodes are basic elements (i.e. proteins, atoms, etc…) and edges model relations among them. In these domains, graph searching plays a key role. For example, in computational biology locating subgraphs matching a specific topology is useful to find motifs of networks that may have functional relevance. In drug discovery, the main task is to find novel bioactive molecules, i.e., chemical compounds that, for example, protect human cells against a virus. One way to support the solution of this task is to analyze a database of known and tested molecules with the aim of building a classifier which predicts whether a novel molecule will be active or not. Future chemical tests can focus on the most promising candidates. Users may ask to find molecules containing the query graph (exact search) or subgraphs similar to the one described by the query (approximate querying) (see Figure 1 for an example).

The graph searching problem can be formalized as follows. Given a database of graphs *D* = {*G*_1_, *G*_2_,…, *G*_n_} (e.g. collection of molecules, etc.) and a query graph *Q* (e.g pattern), find all graphs in *D* containing *Q* as a subgraph. Moreover, all occurrences of *Q* in those graphs should be detected. In many important application *D* consists of a single huge graph. Since most of these problems involve solutions of the graph isomorphism problem, an efficient exact solution can not exists. In order to make searching time acceptable research efforts have tried to improve the following steps [1,2].

1. *Reduce the search space by filtering*. For a database of graphs a filter limits the search to only possible candidate graphs. For a single-graph database only the possible candidate subgraphs are identified. The common idea is to extract structural features of graphs and store them in a global index. When a query graph is presented, its own structural features are extracted and compared with the features stored in the index to check compatibility [3-6]. Most existing systems use subgraphs of small size (typically not larger than 10 nodes). However, even though small subgraphs are used, the size of the index and its time construction may be high. Therefore, high-support/high-confidence mining rules are used to index only frequent and not redundant subgraphs (i.e. a subgraph is redundant when its presence in a graph can be predicted by the presence of its subgraphs) [7-9].

2. *Store Data*. In order to scale to very large databases of graphs indexing structures and data must be stored in secondary memory. Applications make use of advanced database management systems [4].

3. *Match*. After candidate graphs have been selected, an exhaustive search on these graphs must be performed. This step is implemented either by traditional (sub)graph-to-graph matching techniques [10,11] or by an implementation on an extension of the SQL algebra [12].

In this paper, GraphFind, an enhancement of the application-independent graph searching system GraphGrep [5,12], is presented. Experiments show that GraphFind outperforms the compared systems on a very large collection of small molecules, available at the web site of the National Cancer Institute [13]. A key feature of GraphFind is the use of low-support data mining technique (Min-Hashing [14]) to reduce the index size. It is shown that such a mining technique can be successfully applied to enhance other systems such as gIndex [7].

## Results and discussion

### Approach

GraphFind locates all exact and approximate occurrences of a query graph in collections of graphs. It combines filtering techniques described in [5] with a recent matching algorithm [11]. Each graph is stored as a set of small subgraphs. At query time, such a representation allows the selection of candidate subgraphs. GraphFind is implemented on top of Berkeley DB [15] to store both indexing structures and data graphs. A low-support data mining technique (Min-Hashing [14]) is applied to reduce the index size of GraphFind and gIndex [7].

### Related compared systems

GraphGrep [5,12] finds all exact and approximate occurrences of a query graph in collections of graphs. Approximate queries are special subgraphs that may contain: (a) nodes with a special wildcard symbol “?”, that can match any node; (b) approximate paths (represented by a wildcard symbol “*”) which are paths of any length that can connect two nodes. GraphGrep enumerates all small subgraphs (say paths with no more than 4 nodes) in the database together with all occurrences sites and the number of such occurrences. Matching is performed by combining such occurrences making use of an extension of the classical SQL algebra [12].

Daylight [3] is a commercial system to search in molecules databases. The index of each graph is a fixed-size bit vector. It enumerates all existing small paths in a graph, hashes them, and adds them to the vector. A disadvantage of such an approach is that different and unrelated paths may “collide” at the same bit position. An academic freely available emulation of Daylight, called Frowns [10], makes use of an efficient matching subgraph algorithm [11]. All above systems are designed to optimize the query time, at the cost of large preprocessing time.

Data mining techniques have been applied to reduce index construction (space and time) complexity. Related recent work includes [8,9] (stable release of such software are upcoming). gIndex [7] represents the state of art in this area. The key ideas of gIndex are: (i) index frequent subgraphs with a size-increasing support function; (ii) represent them in a canonical form (strings); and (iii) store such strings in a prefix tree.

Although there is a long history of research on indexing for exact searching in database of graphs, only recently have indexing structures for approximate search been proposed [16-18]. SAGA [18] appears to be the most flexible system. It finds subgraphs of a query which are similar (allowing node gaps, node mismatches and graph structural differences) to subgraphs in the database. Algorithms for networks alignment [19] such as NetworkBlast [20] may be used to find approximate occurrences of a query path in a single graph. The main difference between those systems and GraphFind is that GraphFind users may specify precisely at query construction time which nodes or paths are approximate. Thus, GraphFind can not be compared with those systems because it controls the semantics of the output precisely.

### Results

In order to evaluate the performance of GraphFind, we have compared it with the main graph search systems (GraphGrep [12], GFrowns, and gIndex [7]). GFrowns is an implementation of the system Frowns [10] to deal with general graphs. Experiments show that GraphFind compared had better behavior than gIndex in terms of scalability on the tested databases. In addition, GraphFind improves our previous system GraphGrep which is commonly used in the literature as a test system. Experimental analysis was performed on a Pentium IV with 1GB of memory using Linux OS. All algorithms were implemented in C++.

### Test sets

To test the proposed system, a database of 40000 molecules, available at the web site of the National Cancer Institute [13], was used. It contains sparse graphs having from 20 to 270 nodes. The database was divided into subsets of size ranging from 1000 to 40000 molecules.

Systems were tested using a set of 40 queries drawn from the molecules database. The number of nodes, for each query, ranges from 4 to 32. Query time is given as the sum of filtering time and matching time.

Experiments on a single graph database were performed using synthetic data described in [21]. The Min-Hashing technique was analyzed using both synthetic and molecules database.

### Comparisons

**Figure 2:**
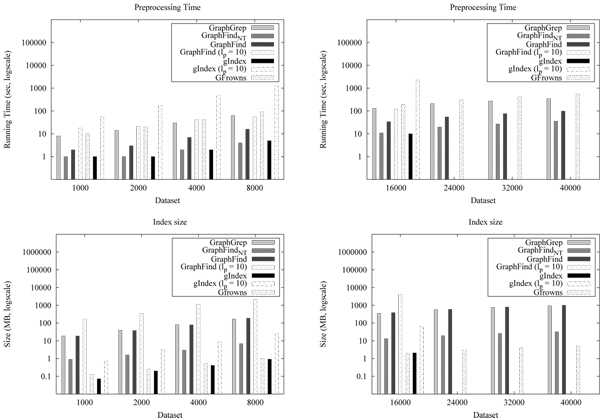
**Preprocessing time and index size for molecular databases.** For all systems the value of *l_p_*, unless specified, is 4. Index size refers to the space needed by the fingerprint database and the data storage. For GraphFind the size of its fingerprint is explicitly indicated (GraphFind_*NT*_). GraphGrep requires the same space as GraphFind. For gIndex and GFrowns data storage is very small compared to its fingerprint size. Preprocessing represents the time to compute the fingerprint and to store all data in secondary memory. Preprocessing of GraphFindNT includes the time needed to compute and write the fingerprint only.

**Figure 3:**
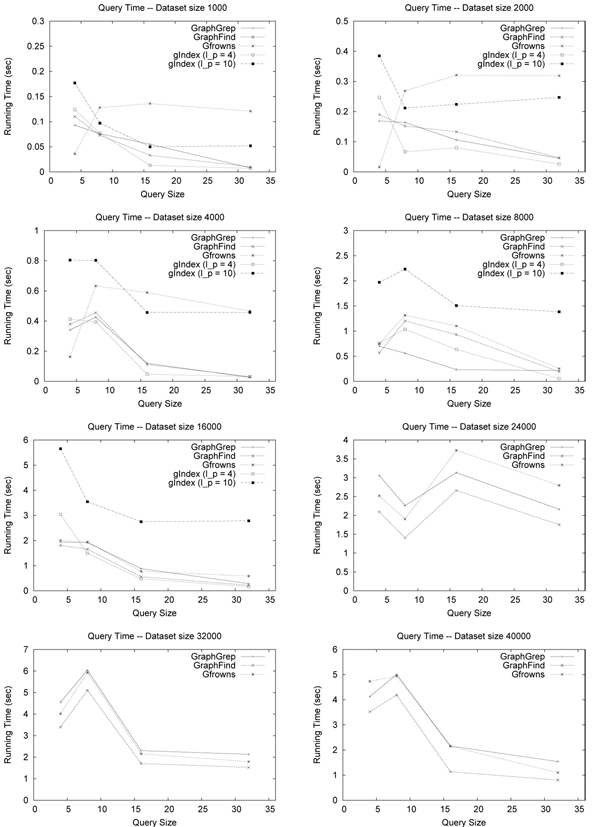
**Query time on molecules database.** Query time: filtering + matching time. GraphFind is scalable compared to gIndex. Querying is comparable. GraphFind outperforms GraphGrep and GFrowns.

Figure 2 reports preprocessing time and index size of GraphFind, GraphGrep, GFrowns, and gIndex on the molecule databases. Concerning *l_p_* = 4, GraphFind and gIndex were comparable and faster than the other systems. However, GFrowns and gIndex outperformed the others on index space. Notice that, the maximum database size treatable by gIndex was 16000. Figure 3 reports querying time. gIndex and GraphFind showed comparable behavior.

Preprocessing time and index size of GraphFind using *l_p_* = 10 is considerable higher than the ones obtained using *l_p_* = 4 (see Figure 2). Results of GraphGrep and GFrowns with *l_p_* =10 are not reported since they are clearly outperformed by GraphFind and gIndex. The querying time of GraphFind (*l_p_* = 10) is not shown since it does not yield any speed-up with respect to the case of *l_p_* = 4.

gIndex filtering with *l_p_* = 10 compared to *l_p_* = 4 discards more graphs, but is slower.

**Figure 4:**
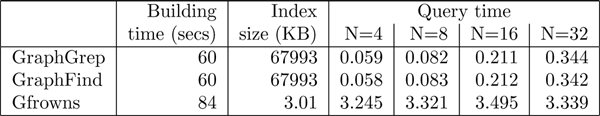
**Comparisons on a single graph database.** Preprocessing and Querying performances of GraphFind, GraphGrep and GFrowns on a single graph database. The graph is a Irregular 3D with 10000 nodes and 5 labels [21]. Index Size refers to the fingerprint database matrix and the graph representation. N is the number of nodes in the query. The query time is the average obtained by 10 different runs. gIndex is not reported since it does not treat graphs with thousands of nodes.

In Figure 4, the performances of GraphFind, GraphGrep and GFrowns on a single large graph are shown. Although the amount of space required by GraphFind is high, the querying results very efficient. gIndex is not reported since it does not treat graphs with thousands of nodes.

**Figure 5:**
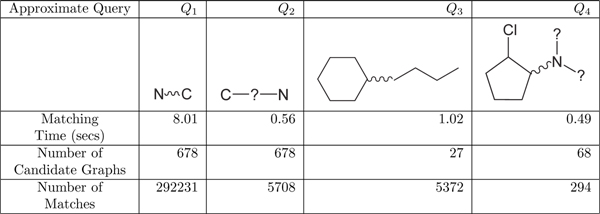
**Running time of approximate queries on a molecules database.** The database contains 8000 molecules, it is available at [13]. Nodes labeled with a special wildcard symbol “?” can match any label; a wavy segment indicates an approximate path of any length (here the wildcard “*” representing an approximate path has been substituted with a wavy segment only for a graphical purpose). Query *Q*_1_ searches for a node (atom) C and a node N connected through a path. Query *Q*_2_ searches for a node (atom) C and a node N connected through a single node. As expected, the number of occurrences of *Q*_1_ is greater than those of *Q*_2_. In each graph, a query may have a large number of occurrences (in 27 candidate graphs, *Q*_3_ has 5372 matches).

Figure 5 reports the performance of GraphFind on approximate queries on a database of 8000 molecules available at [13]. As expected, by increasing the allowed degree of approximation in a query, the execution time and the number of matching subgraphs returned by such a query grow.

**Figure 6:**
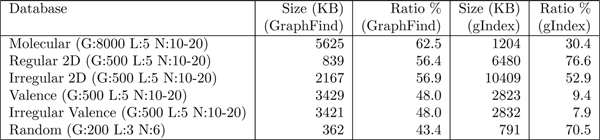
**Index size compression**. Index size compression ratio obtained by applying the Min-Hashing technique on GraphFind and gIndex using real and synthetic databases of graphs. Size indicates the dimension of fingerprint before applying Min-Hashing algorithm. G = number of graphs; L = number of different node labels per graph; N = number of nodes per graph. A description of the synthetic graphs used here is given in [21].

Finally, the Min-Hashing algorithm was applied to reduce the index size of GraphFind and gIndex (see Figure 6). The running time of Min-Hashing does not affect the preprocessing performance (less than one percent of the total time in all tests). However, the index size is considerably reduced in both GraphFind and gIndex.

## Conclusions

This paper has presented GraphFind, an application-independent graph searching system that enhances GraphGrep. The system allows exact and approximate graph searching where the approximations can be precisely specified. Comparisons with competitive systems show that GraphFind performs well and scales better. GraphFind significantly reduces data storage with respect to GraphGrep overhead thanks to low-support data mining. The proposed low-support mining technique, which applies to other searching methods also, reduces indexing space significantly.

GraphFind can be easily implemented in a distributed environment. The database of graphs may be distributed among several servers according to a graph similarity criterion. When graph searching is applied to a huge graph (network), the graph may be partitioned into components based on a minimum cut strategy (e.g. locate hubs and cut at them). Future work will include the design and the experimental analysis of a GraphFind distributed version on web-scale databases. Moreover, methods to rank outputs will be added on specific domains of application. This will be a domain-specific extension. Datasets, software and results are freely available at [22].

## Methods

GraphFind models the nodes of data graphs as having an identification number (*node-id*) and a label (*node-label*). An *id-path* of length *n* is a list of *n* + 1 node-ids with an unlabeled edge between any two consecutive nodes. A *label-path* of length *n* is a list of *n* + 1 node-labels. Label-paths and the id-paths of the graphs in a database are used to construct the index of the database and to store the data graphs.

### Index construction

**Figure 7:**
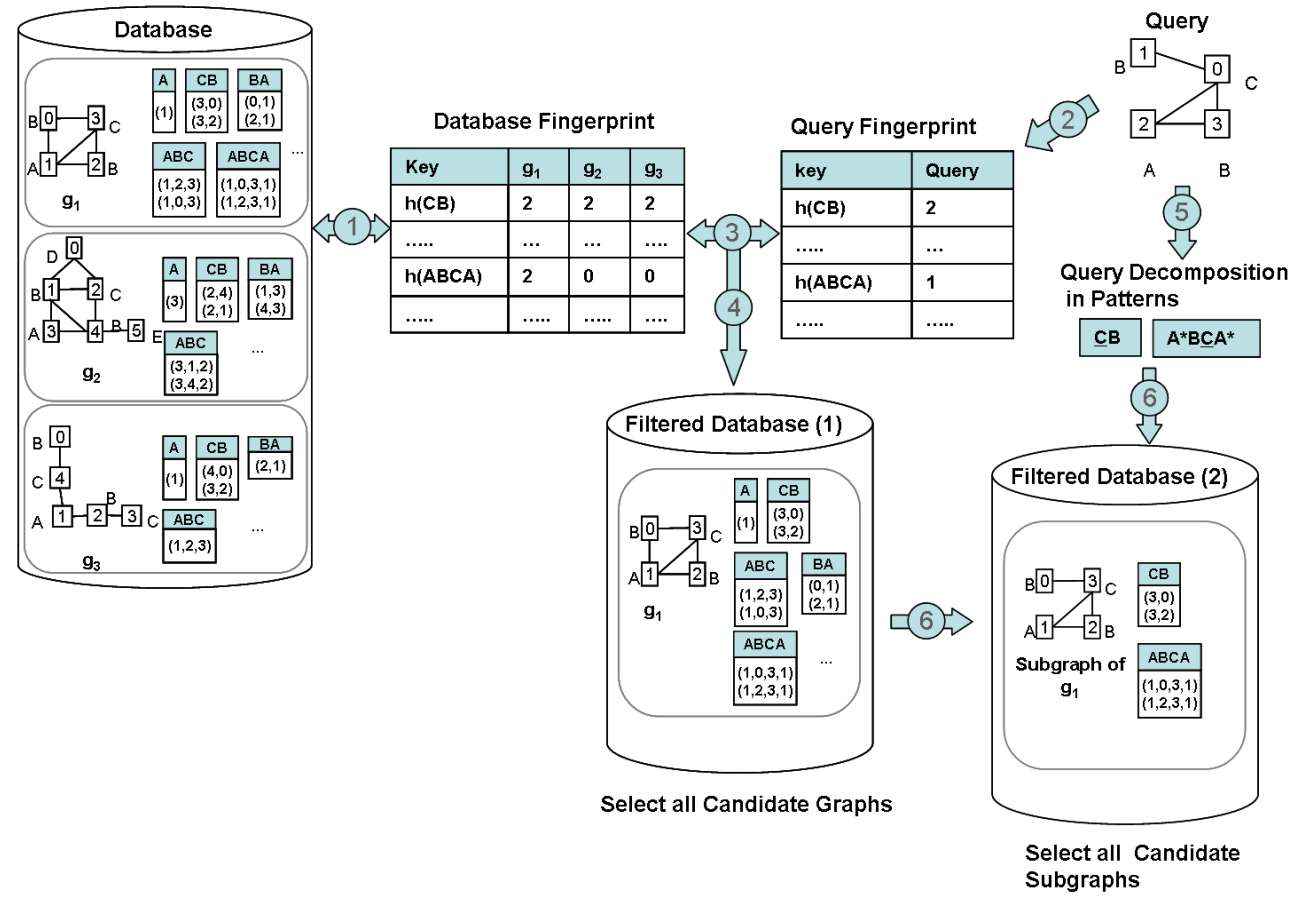
**GraphFind system. Preprocessing** (Step 1): Store each graph in the database in a set of Berkeley DB tables each corresponding to a label-path-set; that is the set of the id-paths (e.g. (3,0), (3,2) in *g*_1_) of all the paths representing a label sequence (e.g. CB in *g*_1_). For each graph only some label-path-sets are shown. The maximum length (number of edges) of a label-path is *l_p_*=3. The fingerprint (index) of a database is a Berkeley DB hash table where each entry in a column is the number of occurrences of a label-path in that graph. **Querying:** Construct the query fingerprint (*l_p_*=3)(Step 2). Compare the fingerprint of the query with the database fingerprint (Step 3): a database graph, for which at least one value in its fingerprint is less than the corresponding value in the fingerprint of the query, is filtered out (Step 4). *g*_2_ and *g*_3_ are not selected as candidates since they do not contain the path ABCA. Decompose the query into patterns (Step 5) (*l_p_*=3). From each candidate graph, select the label-path-sets corresponding to the patterns in the query (Step 6) and combine the id-paths of such tables following the query decomposition criteria. In the patterns (CB, A*BCA*), only labels with equal marks (e.g. _, *) represent the same node occurrences. For example, (1,0,3,1) can not be combined with (3,0) because the nodes labeled B must be different (same motivation applies to (1,2,3,1) and (3,2)). The subgraph obtained by combining (1,2,3,1) and (3,0) is shown in “Filtered Database (2)”. They are the only subgraphs that may match the query. Subgraph matching will be performed by applying the VF2 algorithm [11] to those subgraphs instead of to the entire graphs.

Let *l_p_* be a fixed positive integer. For each graph in the database and for each node, all paths that start at this node and have length from one up to *l_p_* are collected. The index is implemented using a hash table. The keys of the hash table are the hash values of the label-paths. Collisions are resolved by chaining. This hash table is referred as the *fingerprint of the database*. Each entry in a column is the number of occurrences of a label-path in that graph (see Figure 7).

### Data storage

Since several paths may contain the same label sequence, the id-paths of all the paths representing a label sequence are grouped into a label-path-set. GraphFind uses Berkeley DB [15] as the underlying database to store data graph representation and index. GraphFind stores each *fingerprint* as a dynamic Berkeley DB hash table of linked lists (whose keys and values are described above). Each graph is stored in a set of Berkeley DB tables each corresponding to a label-path-set (see Figure 7).

### Queries

A query is an undirected labeled graph. Approximate queries are special subgraphs that may contain: (a) nodes labeled with a special wildcard symbol “?”, which can match any label; (b) approximate paths (represented by a wildcard symbol “*”) which are paths of any length that can connect two nodes.

### Database filtering

The database is filtered by comparing the fingerprint of the query with the fingerprints of the graph in the database. *A database graph, for which at least one value in its fingerprint is less than the corresponding value in the fingerprint of the query, is filtered out*. The remaining graphs are candidates for matching (see Figure 7 (Filtered Database(1))). Next, parts of the candidate graphs are filtered out as follows: (i) decompose the query into patterns and (ii) *select only those id-path sets associated with patterns in the query* (see Figure 7 (Filtered Database(2))). The selected id-path sets correspond to one or several subgraphs of candidate graphs. Those subgraphs are the only ones that may match the query.

### Subgraph exact and approximate matching

After filtering, subgraph matching on the possible matching candidates is performed by applying the VF2 algorithm [11] to each candidate. This is a refinement of Ullmann's subgraph isomorphism algorithm that uses more selective feasibility rules to prune the state search space. Approximate queries are handled by independently processing, as described above, all maximal exact (completely specified) subqueries. The resulting subgraph matchings are then “joined” by checking, for each pair of query nodes connected by an approximate path, if there is a path in the data graph (of length equal to the wildcards' values) between the corresponding matched nodes. This is performed by using depth-first search. As shown in [11], the computational complexity in the worst case of the VF2 algorithm is Θ(*N!N*), where *N* is the number of nodes in the query.

### Indexing by low support data mining techniques

Let *M*(*m*,*n*) be the fingerprint of a graph database. Rows correspond to graphs, columns are patterns and each entry is the number of occurrences of each pattern in that graph. Two patterns are similar if a large number of graphs have the same number of occurrences of it. More precisely, let the similarity *Sim*(*C_i_*, *C_j_*) of two columns be the percentage of non null rows in which the two columns have the same value. The aim of the Min-Hashing algorithm [14] is to quickly find pairs of columns (indexed patterns) that have a similarity greater than a given threshold s*. It generates *k* random permutations, say *p^j^* : {1,…,*m*} → {1,…,*m*} for *j* = 1,···,*k*, of row indices of *M*. $p_{i}^{j}$ denotes the *i*-th element of the permutation *p^j^*. Let $\bar{M}\left(k,n\right)$ be the corresponding *signature matrix* of *M*. Each entry $\bar{M}\left[i,j\right]$ is the index *t* of the first row in *M* in which $M\left[p_{t}^{i},\;\;j\right]\neq 0$. Formally, $\bar{M}\left[i,j\right]$ =*t* if and only if $M\left[p_{t}^{i},j\right]\neq 0$ ∀*s* <*t*, $M\left[p_{s}^{i},j\right]=0$. Let the similarity *Sim*(*C_i_*, *C_j_*) of two columns *C_i_* and *C_j_* be defined as $\frac{\left|C_{i}\cap C_{j}\right|}{\left|C_{i}\cup C_{j}\right|}$. In [14] the authors show that the similarity of two columns is well approximated by the similarity of the corresponding columns in the signature matrix. Consequently, finding similar columns in the matrix becomes a lightweight computation. This allows deletion of columns which are similar to others. Such a technique can be applied to any indexing system. In GraphFind it is applied to the transposed database fingerprint matrix (see Figure 7). Moreover, in GraphFind s* is not a user parameter. The system is designed to find pairs of columns (patterns) with similarity s* = 100% in the fingerprint database. Therefore, two patterns that have the same occurrence in each graph will be represented in the matrix using only one column indexed by both patterns. Notice that, by reducing the similarity threshold s*, correctness is maintained and the compression ratio may be higher. However, this implies a loss in filtering efficiency and therefore greater searching time. Figure 6 reports the compression ratio of index size in both GraphFind and gIndex after Min-Hashing.

## List of abbreviations used

3D: Three-Dimensional

DB: Database

G : Number of Graphs

GB: Gigabyte

GraphFind_NT_: GraphFind Fingerprint

GFrowns: Graph Frowns, Implementation of Frowns for General Graph

L : Number of Different Node Labels

*l_p_*: Length of Label Path

OS: Operating System

N: Number of Nodes

SQL: Structured Query Language

SAGA: Substructure Index-based Approximate Graph Alignment

VF2: Graph Matching Algorithm by Vento Foggia et al.

## Competing interests

The authors declare that they have no competing interests.

## Authors' contributions

All authors designed, analyzed, implemented and tested the proposed algorithm. Each author contributed equally in writing the paper. All authors read and approved the final manuscript.
